# A Novel Pathway of Flavonoids Protecting against Inflammatory Bowel Disease: Modulating Enteroendocrine System

**DOI:** 10.3390/metabo12010031

**Published:** 2022-01-01

**Authors:** Mingrui Li, Benno Weigmann

**Affiliations:** 1 Department of Medicine 1, Kussmaul Campus for Medical Research, University of Erlangen-Nürnberg, 91052 Erlangen, Germany; Li.Mingrui@extern.uk-erlangen.de; 2 Medical Immunology Campus Erlangen, Friedrich-Alexander University Erlangen-Nürnberg, 91052 Erlangen, Germany

**Keywords:** inflammatory bowel disease, flavonoids, GLP-1, GLP-2, DPP-4 inhibitors, ghrelin, cholecystokinin (CKK)

## Abstract

Inflammatory bowel disease (IBD) is a comprehensive term for chronic or relapsing inflammatory diseases occurring in the intestinal tract, generally including Crohn’s disease (CD) and ulcerative colitis (UC). Presently, the pathogenesis of IBD is unknown, yet multiple factors have been reported to be related with the development of IBD. Flavonoids are phytochemicals with biological activity, which are ubiquitously distributed in edible plants, such as fruits and vegetables. Recent studies have demonstrated impressively that flavonoids have anti-IBD effects through multiple mechanisms. These include anti-inflammatory and antioxidant actions; the preservation of the epithelial barrier integrity, the intestinal immunomodulatory property, and the shaping microbiota composition and function. In addition, a few studies have shown the impact of flavonoids on enterohormones release; nonetheless, there is hardly any work showing the link between flavonoids, enterohormones release and IBD. So far, the interaction between flavonoids, enterohormones and IBD is elucidated for the first time in this review. Furthermore, the inference can be drawn that flavonoids may protect against IBD through modulating enterohormones, such as glucagon-like peptide 1 (GLP-1), GLP-2, dipeptidyl peptidase-4 inhibitors (DPP-4 inhibitors), ghrelin and cholecystokinin (CCK). In conclusion, this manuscript explores a possible mechanism of flavonoids protecting against IBD.

## 1. Introduction

Inflammatory bowel disease (IBD) is a general term for chronic or relapsing inflammatory diseases occurring in the intestinal tract, mainly including Crohn’s disease (CD) and ulcerative colitis (UC). In addition to CD and UC, IBD also contains IBD unclassified (IBDU) [1]. CD is a main form of IBD, which may affect any region of the gastrointestinal tract. In general, the symptoms are comprised of weight loss, abdominal pain, diarrhoea, fever and abdominal distension [2]. In contrast, UC is another major form of IBD, which starts in the rectum and generally extends proximally in a continuous manner through part of, or the entire, colon. Bloody diarrhoea is the characteristic symptom of the disease [3]. Over the past decades, the prevalence of IBD has been significantly becoming higher in the world and entails a huge economic burden to both patients and countries [4]. Although the pathogenesis of IBD is unclear nowadays, etiological studies have shown that the development of IBD is associated with genetic susceptibility, environmental factors, intestinal microbiota and immunological abnormalities [5]. Flavonoids are a group of plant chemicals found in almost all vegetables and fruits. A large number of studies indicate that an everyday intake of flavonoids produces multiple health benefits to the human body, such as a decreased risk of numerous chronic diseases involving cardiovascular diseases and cancer [6]. In recent years, it was found that flavonoids can directly treat IBD through various mechanisms, including anti-inflammatory and antioxidant actions, which preserve the epithelial barrier, and immunomodulatory functions in the intestine, which shape the composition and function of the microbiota [7,8]. For instance, Dryden et al. [9] investigated the safety and efficacy of epigallocatechin-3-gallate (EGCG) on UC patients and found that EGCG belonging to the flavonoid family obviously decreased the disease activity index score of UC patients compared with the placebo group. Moreover, flavonoids have been reported to be able to modulate the enterohormone secretion [10,11]. Currently, very few papers elucidate the relevance between flavonoids, enterohormones and IBD. Based on a few studies suggesting that enteroendocrine cells and their secretory hormones play an influential role in the development of IBD [12,13,14], it is effortless to hypothesise that flavonoids may protect against IBD via a possible pathway—namely, the modulation of the enteroendocrine system. In order to bring some comprehensive insight into the modulatory system, this article will now explore a possible mechanism of flavonoids protecting against IBD.

## 2. Flavonoids: Classification, Metabolism, Absorption and Bioavailability

Flavonoids are ubiquitous in the vegetable kingdom and consumed in the human diet. As a group of phytochemicals, the structure of flavonoids is comprised of two benzene rings that are linked by a pyran heterocyclic ring [15]. Currently, more than 4000 varieties of flavonoids have been identified and determine the bright colours of diverse fruits and vegetables [16]. Based on their classes, some dietary sources of flavonoids are classified in Table 1 [17].

Flavonoids mainly exist in the forms of aglycones and glycosides, as well as methylated derivatives, among which aglycones are the basic flavonoid structure [18]. Nevertheless, glycosides are the main form of flavonids that exist in plants [19]. The physicochemical characters of dietary flavonoids determine their absorption, such as solubility, molecular size, configuration and lipophilicity, as well as pKa [18]. For instance, glycosides hardly penetrate the cell membrane because of their hydrophilicity. As a result, glycosides can be absorbed only through the hydrolysis of sugar conjugates or a special active transport pathway in the gut [20].

In the small intestine, aglycones can be easily absorbed by passive diffusion. Contrastingly, glycosides are not assimilated and absorbed and, thus, arrive at the colon and must be converted into an aglycone form by the colonic microflora and simultaneously break up the released aglycones into small molecule compounds, which continue to be degraded or absorbed in the large intestine [18,19,20]. Some flavonoids are also metabolised back into the small intestine through bile secretion and release. Free aglycones are neither presented in the plasma or in the urine, since some conjugation reactions happen, with the exception of catechins [17]. Metabolism can occur in the liver and the kidney through blood circulation [21]. Through the process of flavonoids metabolism, it is discovered that the intestine is exposed to the highest concentration of flavonoids in the human body.

## 3. Canonical Mechanism of Flavonoids Regulating IBD

The biological activities of flavonoids encompass immune adjustment, antioxidants, scavenge free radicals, the changing of the microbiota constitution, anticancer effects and the restoring of damaged epithelial barriers. Especially, the properties of antioxidant and immune regulation can inhibit inflammation. For this reason, flavonoids are capable of mitigating the inflammatory level of IBD [22]. For the first time, Galsanov et al. [23] (1976)_discovered that flavonoids have a considerable potential to relieve inflammation in the intestine. They found that quercitrin has a strong anti-inflammatory activity in rat models with allergic intestinal inflammation. Afterwards, a considerable amount of literature was published on the effect of the flavonoids in colitis rodent models [17]. These studies show that diverse flavonoids are able to inhibit inflammation, comprising those of different chemical classes. At present, the canonical mechanisms of flavonoids treating IBD have been proposed, including antioxidant action, the protection of the epithelial barrier integrity, the regulation of the immune function in the intestine and the positive shaping of the microbiota composition and function [8].

### 3.1. Antioxidant Property

It has previously been observed that reactive nitrogen (RNS) and oxygen (ROS) species take part in the development of IBD [24]. Some studies showed that there is an overproduction of RNS and ROS in the gut of IBD [25]. Previous studies established that mononuclear cells and neutrophiles invade the damaged portion of the intestine in patients with IBD, in which the nicotinamide adenine dinucleotide phosphate (NADPH) oxidase system is activated, accordingly inducing myeloperoxidase (MPO) activity. With the combined action of NADPH and MPO, a large amount of superoxide, as well as hypochlorous acid, is produced. Correspondingly, cytotoxicity is directly produced in the affected parts of the intestine [26]. On the other hand, this approach causes an overproduction of diverse proinflammatory factors [27]. Nevertheless, recent studies through in-vivo experiments have confirmed that most flavonoids can obviously decrease MPO in the colon, which is considered as a critical marker of leukocyte invasion [28]. Additionally, most flavonoids are capable of reducing the production of RNS and ROS in colitis models [29].

Under the action of the nitric oxide synthase (NOS) enzyme, L-arginine is broken down into nitric oxide (NO), which is considered a pleiotropic free radical messenger molecule. The isoform of constitutive NO synthase (cNOS) generates a small amount of NO in the physiological environment. At the beginning of the intestinal inflammation, NO is able to directly protect against the destruction of inflammation. In contrast, the expression of the inducible isoform of NO synthase (iNOS) significantly increases in chronic inflammation, leading to the upregulation of NO synthesis [30], which causes an overproduction of NO. After interacting with the superoxide anions, excessive NO generates an enormous amount of peroxynitrites, which contribute to the intestinal damage [31]. Extensive research has shown that flavonoids can exercise lots of influence on NO metabolism [32], such as the inhibition of the expression of iNOS [33]. As a consequence, flavonoids are able to mitigate the enterotoxicity of NO, which is excessively produced during intestinal inflammation.

### 3.2. Preservation of Epithelial Barrier Functon

The intestinal epithelial barrier mainly consists of a single layer of enterocytes, which are located between the gut lumen and host tissue, playing a critical role in the maintenance of gastrointestinal homeostasis. The impairment of the intestinal barrier function has been found in many patients with IBD. A few studies have shown that an abnormal function of the epithelial barrier exists at the onset of intestinal inflammation. It is caused by the infiltration of pathogens from the intestinal lumen to the host tissue, prompting disorder of the immune system [34,35]. Interestingly, a certain amount of studies have indicated that flavonoids have a beneficial effect on intestinal inflammation, including in vivo and in vitro experiments. In 2013, Azuma et al. [36] reported that naringenin decreased the permeability of the damaged epithelial barrier, induced by dextran sodium sulphate (DSS) administration, which is completed by preserving the barrier function and the structure of the intestinal tight junction. Cell experiments have also testified about the protective effects of flavonoids on the epithelial barrier function. Noda et al. [37] incubated human intestinal Caco-2 cells with different varieties of flavonoids. They found that daidzein, hesperidin, naringenin and morin enhanced transepithelial electrical resistance (TEER), indicating an increased tight junction integrity, which confirmed that flavonoids are capable of protecting the epithelial barrier function.

### 3.3. Immunomodulatory Property in the Gut

The abnormal immune response plays a vital role in the pathogenesis of IBD, which can increase the synthesis and secretion of proinflammatory cytokines like interleukin 1β (IL-1β), interleukin 6 (IL-6) and adhesion molecules, as well as chemokines. Diverse in vivo studies have shown that flavonoids are able to modulate the abnormal immune response occurring in gut inflammation. For example, Bruckner et al. [38] reported that epigallocatechin gallate (EGCG) significantly reduced the production of proinflammatory cytokines in murine colitis models induced by an oral administration of DSS. Many in vitro studies have also confirmed the immunomodulatory character caused by flavonoids. For instance, Mascaraque et al. [39] found that rutin has an anti-inflammatory activity in chronic colitis models through inhibiting the plasma level of proinflammatory cytokines, involving interferon γ (IFN-γ) and tumour necrosis factor (TNF-α).

Among all the immune cells, T cells are the major participants in the development of IBD [40]. In addition, the proportion of Th1 and Th17 cells in mesenteric lymph nodes increase in colitis models induced by DSS, leading to an excessive production of proinflammatory cytokines. In vitro studies have proven that flavonoids are capable of inhibiting the activation and proliferation of T cells. Likewise, macrophages are another major origin of the proinflammatory factors in IBD. Additionally, in vivo studies have also uncovered that flavonoids have anti-inflammation properties. For instance, it was reported by Camuesco et al. [33] that quercitrin remarkably decreased the amount of macrophages infiltrated in the inflamed murine colon induced by DSS. Furthermore, one of the main pathological events in IBD patients is neutrophil infiltration [26]. In vivo studies have suggested that most flavonoids are able to notably decrease the extent of neutrophil infiltration in the affected colon so as to ameliorate the intestinal inflammation [41].

Other studies pay attention to the modulatory mechanisms of cytokine production, some of which correlate with the suppression of mitogen-activated protein kinase (MAPK) and nuclear factor-κB (NF-κB), as well as signal transducer and activator of transcription (STAT) activation. Most flavonoids are able to block the above pathways. In this way, the level of inflammation in IBD is suppressed [8].

### 3.4. Shaping Microbiota Composition and Function

Dysbiosis usually occurs in patients with IBD, in which the microbiota in the gut shows reduced biodiversity (mainly Firmicutes); diminished stability and enlargement of Proteobacteria (*Enterobacteriaceae*, *Bilophila* and some members of *Bacteroidetes*) [42]. A reduction or loss in biodiversity may contribute to a loss or diminution of the key functions that are indispensable for modulating the intestinal immune system and sustaining the enteroepithelial barrier integrity, probably leading to inflammation and abnormal immune responses in the intestine [43]. Based on antimicrobial and antioxidant characters, diets rich in flavonoids have the potential to be the complementary remedies for IBD [44]. In this context, some studies have showed that alteration of the microbiota constitution induced by flavonoids exerted a beneficial influence on gut inflammation. For instance, Hong et al. [45] investigated the impact of quercetin on the gut microbiota diversity in colitis mice induced by DSS. They found that a reduced Firmicutes population and an increased Proteobacteria population occurred in DSS-induced mice, but quercetin supplementation can ameliorate these effects, which indicated that quercetin may alter the consequences of microbiota-associated disorders. In addition, Ren et al. [46] discovered that acacetin also could alleviate the alteration of gut microbiota in colitis mice and that Firmicutes were reduced and *Escherichia–Shigella* were increased. Furthermore, *Escherichia–Shigella* have been reported to show a positive correlation with the overexpression of inflammatory cytokines, thus causing adverse effects in IBD. Recently, Wu et al. [47] discovered the microbial-modulating effect of EGCG extracts from green tea in an experimental colitis model. Oral administration demonstrated an accumulation of *Akkermansia* and, consequently, an increased production of SCFA. EGCG could be used in this study as a preventive modulator for the induction of a remission.

## 4. Enteroendocrine System

### 4.1. Enteroendocrine Cells: Subtypes and Functions

**Figure 1:**
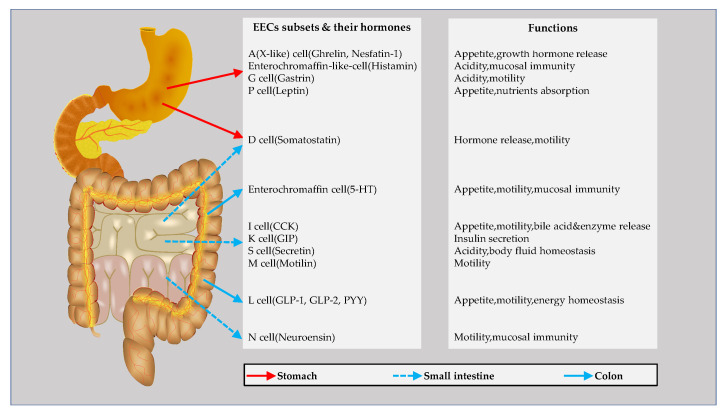
Schematic illustration of enteroendocrine cell (EEC) subsets in the gastrointestinal tract, as well as a demonstration of the major secretary hormones and their functions. 5-HT, 5-hydroxytryptamine; CCK, cholecystokinin; GIP, glucose-dependent insulinotropic peptide; GLP-1, glucagon-like peptide-1; GLP-2, glucagon-like peptide-2; PYY, peptide YY.

The enteroendocrine system is comprised of enteroendocrine cells (EECs), which are specialised cells with an endocrine function, distributed in the gastrointestinal tract, as well as the pancreas, producing gastrointestinal hormones in response to intestinal lumen stimuli, as well as releasing them into the blood stream as a systemic effect. The EECs can also diffuse them as local messengers or transmit them to the enteric nervous system to generate nervous regulation [48,49]. EECs are dispersed in the crypts and villi along the gastrointestinal mucosa, which compose the largest endocrine system in the human body, making up 1% of the intestinal epithelium [14]. In general, EECs are classified into at least 10 categories according to their different secretory hormones (see Figure 1), such as vasoactive intestinal peptide, substance P, cholecystokinin, serotonin, members of the chromogranin/secretogranin family, glucagon-like peptide (GLP), somatostatin and neuropeptide Y (NPY), as well as ghrelin [50]. In addition, enterochromaffin cells, considered as the largest population of EECs, have the capability to produce and release serotonin (5-HT), accounting for more than 95 percent of 5-HT in the whole body [51]. New findings show that a type of EEC is capable of synthesising/releasing two and more hormones. For example, it was reported that glucagon-like peptide (GLP-1) and the peptide YY (PYY) are expressed together in enteroendocrine L cells [52,53]. The EEC-derived hormones exert diverse effects on the human body, such as regulating nutrient absorption and the immune system in the gut and protecting the epithelial barrier and visceral hyperalgesia, as well as colonic motility [54]. As a soluble and heat-stable protein, Chromogranin-A (CgA) is preserved and secreted from the storage granules of EECs. The proteolytic cleavage of CgA produces CgA-derived peptides (CgDPs) in the intestine [55], including pancreastatin (PST), serpinin, vasostatin (VS), catestatin (CST) and chromofungin (CHR) [56,57].

Luminal contents, such as nutrients and microbiota, stimulate the specialised sensory microvilli of EECs so as to release hormones [58]. The release of intestinal hormones packaged into secretory vesicles is controlled by increased concentrations of cytoplasmic calcium, as well as cyclic adenosine monophosphate (cAMP). Only in this condition can EECs detect ingested food, which is macronutrients broken down into their ingredients like glucose and amino acids, as well as fatty acids. Specialised receptors and transporters of EECs detect small compounds so as to trigger the secretion of hormones.

### 4.2. Changes of Enteroendocrine System in IBD

Recent studies have shown that the enteroendocrine system has a pivotal role in the pathogenesis of IBD, which is demonstrated by the alteration of EEC numbers and their secreted hormones in IBD patients and rodent colitis models. For instance, two enteroendocrine markers, ubiquitination factor E4A (*UBE4A*) and paired-like homeobox 2b (*Phox2B*), are reported to significantly augment the distal ileum of patients with Crohn’s disease [59]. Related papers have shown that the amount of colonic PYY and pancreatic polypeptide (PP), as well as the number of oxyntomodulin-producing EECs, were decreased in patients with Crohn’s disease [60]. Salhy et al. [61] set up rat colitis models induced by trinitrobenzene sulfonic acid (TNBS) and found that, compared with the control group, the amount of PYY, CgA and pancreatic polypeptide-producing cells obviously decreased in the TNBS group, contrary to the number of serotonin and oxyntomodulin, as well as somatostatin-producing cells, significantly increased in the TNBS group. Furthermore, Salhy et al. [62] induced colitis models by DSS in rats. They discovered that the amount of CgA, serotonin, PYY and oxyntomodulin cells got higher, but the number of pancreatic peptide and somatostatin cells got lower in DSS-induced rats than in the controls. The above studies indicated EECs and their secreted-hormone responses to colitis in rats. Strid et al. [63] reported that the serum and plasma CgA in IBD patients significantly increased; however, the CgA levels in stool only increased in patients with ulcerative colitis, which did not correlate with disease severity. CgA and its derivatives like VS and CST, have been proven to be able to modulate antimicrobial property, which suggests that the alteration of CgA in EECs relates to the variation of the microbial composition, richness and diversity in the gut [64,65]. Other enterohormones such as CCK, GLP-1, 5-HT, motilin and gastrin also change during the development of IBD [66,67,68,69,70,71,72].

### 4.3. Flavonoids Regulate the Enteroendocrine System

**Figure 2:**
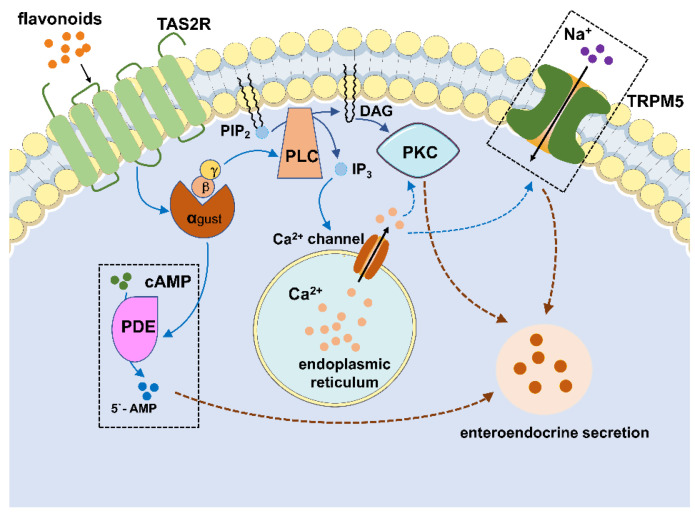
Potential pathways of enteroendocrine hormone secretion induced by flavonoids. The combination of ligands and TAS2R stimulates a signalling cascade, which comprises the G-protein gustducin detaching from the Gα and Gβγ subunits, activating phospholipase C β2 (PLCβ_2_) and producing diacylglycerol (DAG) and inositol 1, 4, 5-trisphophate (IP_3_), as well as opening transient receptor potential ion channel M5 (TRPM5), releasing intracellular Ca^2+^ ([Ca^2+^]_i_), Na^+^ influx, cellular depolarisation and the secretion of neurotransmitters. DAG and [Ca^2+^]_i_ also activate the protein kinase C (PKC) pathway. Additionally, the increased level of the intracellular Gα subunit triggers phosphodiesterase.

Taste receptors and gustatory proteins are expressed in the gut, as well as participate in the chemosensation of nutrients. Taste receptors on EECs can identify and bind to nutrients and beneficial compounds (like flavonoids) in the intestine and take part in the process of nutrient absorption and the regulation of glucose homeostasis [73]. Taste receptors are cellular receptors that have the function of sensing tastes. Up to now, there have been five basic tastes identified (salty, sweet, bitter, sour and umami). Salty and sour tastes are both detected through the pathway of ion channels, and the rest of the tastes (sweet, bitter and umami) are detected by taste receptors [74]. Vertebrate taste receptors are divided into two main families: the taste 1 receptor family, TAS1R, sensing sweet and umami tastes, and the taste 2 receptor family, TAS2R, sensing bitter taste. Most taste receptors are classified into the G protein-coupled receptor (GPCR) family, transducing signals from the intestinal lumen to the inside of the cell [75]. Currently, some studies have discovered that TAS2R involving bitter taste receptors are expressed on enteroendocrine cells and bind to flavonoids, consequently stimulating gut hormones secretion. For instance, Serrano et al. [76] investigated the effects of grape-seed phenolic compounds on ghrelin secretion in rats and found that monomeric flavanols increased ghrelin secretion by binding to bitter taste receptors; however, polymeric forms decreased ghrelin secretion. Several studies also showed a few polyphenols that are able to activate bitter taste receptors, which were operated in silico [77], or based on HEK293 cells expressing bitter taste receptors of human [78]. Therefore, the activation of bitter taste receptors takes a crucial role in gut hormone release [79]. Figure 2 shows the potential mechanism of flavonoids stimulating gut hormone secretion by binding TAS2R [80].

Accumulating studies have begun to enumerate the alteration of gut hormones in EECs stimulated by flavonoids. Gonzalez et al. [81] found that grape seed extract increased the level of plasma GLP-1 in rat models. Kato et al. [82] demonstrated that anthocyanin delphinidin 3-rutinoside stimulated GLP-1 secretion in murine GLUTag cells (a model of EECs). Cremonini et al. [83] observed that epicatechin significantly increased the level of circulating GLP-2 in mice. Cani et al. [84] reported that prebiotics remarkably enhance the plasma level of GLP-2 in mice, whereby there exists a hypothesis that flavonoids stimulate enterohormone production and release through shaping the microbiota composition, which has been stated before. Zhang et al. [85] investigated the effect of soy isoflavones on the weight of rats fed with a high-fat diet. They figured out that soy isoflavones obviously decreased the plasma level of ghrelin, increased the plasma concentration of CCK and also increased the PYY level in plasma with no statistical significance. On the other hand, the similar effect of isoflavones inhibiting ghrelin release has not been presented in human studies. Matvienko et al. [86] designed a group of healthy postmenopausal women who accepted an oral administration of soy isoflavones (1.19–1.79 mg/kg bw) for 12 months. They found that there was no significant difference of the fasting concentration of the plasma ghrelin between the treatment group and the control group. In conclusion, different kinds of flavonoids have disparate effects on ghrelin secretion, since more than 4000 varieties have been identified. During another study with healthy postmenopausal women, PYY was increased by isoflavones [87]. A cell experiment showed that CCK peptide hormone secretion from enteroendocrine STC-1 cells was stimulated by quercetin, kaempferol, rutin, apigenin and baicalein [88]. Another study indicated that catechin/epicatechin increases CCK secretion [89].To sum up, a number of studies confirmed that flavonoids are able to modulate GLP-1/2, Ghrelin, PYY and CCK levels; however, very little is currently known on flavonoids regulating other enterohormones.

## 5. Flavonoids Regulate IBD Mediated by EECs

At present, studies have shown that EECs can modulate IBD through the following pathways: sensing gut microbiota and regulating the immune response, thus protecting the intestinal physical barrier, as well as the regulation of gut motility [55]. As previously mentioned, flavonoids have an influence on the enteroendocrine system. Based on the capability of the flavonoids to protect against IBD, we infer that the alleviation of IBD is possibly related to the modulation of flavonoids on EECs, which take part in the above pathways and can thus regulate IBD. The introduction of different enteroendocrine hormones regulating IBD is discussed in the following sections.

### 5.1. GLPs/DPP-4 Inhibitors and IBD

**Figure 3:**
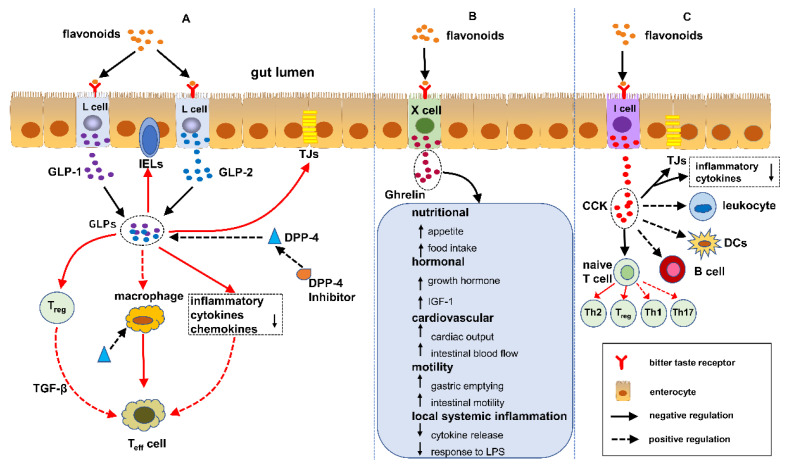
(**A**) Flavonoids regulate IBD by the DPP-4/GLPs pathway: (1) protecting the intestinal barrier, (2) modulating Treg and intraepithelial lymphocytes (IELs) by managing their differentiation and functions and (3) adjusting the function of macrophages and dendritic cells. (**B**) Flavonoids regulate IBD by the ghrelin pathway: (1) increased food intake, (2) increase in growth hormone activity, (3) cardiovascular effects, (4) increased motility and (5) decreased local and systemic inflammation. (**C**) Flavonoids regulate IBD by the cholecystokinin (CCK) pathway: (1) decreasing the mucosal production of proinflammatory cytokines and protecting the intestinal barrier, (2) attenuating leukocyte migration and inhibiting dendritic cells (DCs) activation and (3) regulating T cells and B cells.

GLPs are comprised of GLP-1 and GLP-2, secreted by intestinal L cells. We stated previously that flavonoids are capable of significantly increasing the GLP-1/2 level in plasma. Moreover, flavonoids can suppress the activity of dipeptidyl peptidase 4 (DPP-4), consequently elevating the half-period of circulating GLPs [11]. At present, some studies have shown that GLPs, and the DPP-4 inhibitor have great potential in offering some therapeutic options for IBD. The latent protective pathways likely include: (1) GLPs that facilitate the recovery of the damaged epithelial barrier; (2) GLPs that modulate the differentiation of T cells and their functions; (3) GLPs and DPP-4 that have an impact on the innate immune cells (macrophages, dendritic cells, etc.) and (4) a GLP level that can be improved through inhibiting the enzymatic activities of DPP-4 (see Figure 3A [90]). Below, we outline the essential statements on their roles in IBD. Moreover, these studies are summarised in Table 2.

#### 5.1.1. GLP-1

Recently, mounting evidence has confirmed the anti-inflammatory property of GLP-1. Yusta et al. [91] investigated the effect of GLP-1 on topical inflammation by DSS inducing GLP-1 receptor knockout mice and wildtype mice. Finally, they found that GLP-1 receptor knockout mice achieved higher scores of intestinal inflammation than wildtype mice, which was evaluated by weight loss, disease activity and intestinal epithelial damage. Chassaing et al. [92] investigated the impact of GLP-1 on DSS-induced colitis in C57BL/6 mice, which received an exendin-4 (GLP-1 agonist) injection. They discovered that exendin-4 treatment did not decrease the level of intestinal inflammation compared to the control mice. The reason probably lies in the fact that exposure to exendin-4 is inadequate, since exendin-4 is quickly degraded in mice. Afterwards, Bang-Berthelsen et al. [93] worked on the effect of liraglutide (a long-acting GLP-1 agonist) on intestinal inflammation in severe combined immunodeficiency mice. They found that liraglutide significantly mitigated colitis through decreasing the inflammatory score in histopathology and the level of proinflammatory cytokines. Anbazhagan et al. [94] adopted GLP-1 nanomedicine to treat DSS-induced colitis in mice, which can increase the in vivo stability so as to extend the half-life of GLP-1. They found that GLP-1 nanomedicine obviously ameliorated the inflammation level of colitis, including decreasing the weight loss and improving the stool consistency, as well as alleviating the histological destruction. Furthermore, GLP-1 nanomedicine reduced the expression of IL-1β and increased the expression of intestinal chloride transporter DRA, which can mitigate IBD-derived diarrhoea. Currently, although a large amount of evidence has indicated that GLP-1 has the capability to treat IBD in animal experiments, there are rare human experiments on GLP-1 treatment in IBD. Recently, one paper reported that GLP-1 achieved a satisfactory therapeutic effect in a patient with ulcerative colitis, although there was a lack of adequate samples [95]. Villumsen et al. [13] organised a large-scale epidemiological survey, which suggested that the administration of GLP-1 receptor agonists and/or DPP-4 inhibitors can improve the process of IBD to a great extent. In the survey, they analysed all the data of 3751 patients with IBD and type 2 diabetes together. They were treated by an antidiabetic drug (GLP-1 receptor agonists/DPP-4 inhibitors: 982 patients in total; other antidiabetic treatment: 2769 patients in total). On the whole, we can say with certainty that flavonoids are able to increase the GLP-1 level, which can alleviate gut inflammation and related symptoms, thus achieving the aim of gradually regulating IBD. However, concerning the interaction between the flavonoids, GLP-1 and IBD, there are no direct experimental data to verify it so far.

#### 5.1.2. GLP-2

Compared with GLP-1, the amount of published studies on GLP-2 treating IBD is not much. Recently, some animal experiments have investigated the protective effect of GLP-2 on IBD. Drucker et al. [96] used teduglutide (a GLP-2 analogue) to treat DSS-induced colitis in mice, which was demonstrated to notably increase the weight of the affected small intestine and colon compared to the control mice. Furthermore, the improvement of the histological structure and enhancement of crypt cells proliferation were presented in teduglutide-affected mice. Ivory et al. [97] examined the effect of GLP-2 on colitis models in IL-10 knockout mice. They recognised that GLP-2 injection inhibited weight loss, decreased myeloperoxidase activity and diminished the inflammation score in histopathology in comparison with the placebo group. Furthermore, GLP-2 injection lowered the level of proinflammatory cytokines, such as IL-1β, IFN-γ and TNF-α. These results suggest that GLP-2 has IL-10-independent anti-inflammatory effects. The native GLP-2 in circulation is rapidly degraded. Therefore, the current studies are inclined to focus on the GLP-2 analogue extending the half-life of GLP-2. Yang et al. [98] investigated the anti-inflammatory activity of peptide-10, which has a 10 times longer half-period than teduglutide, the half-life of which is 3–5 h. They discovered that peptide-10 exhibited stronger anti-inflammatory effects than with teduglutide in mice colitis models induced by DSS. Qi et al. [99] set up rat colitis models induced by DSS and observed the anti-inflammatory effect of another GLP-2 analogue (polyethylene glycosylated porcine, GLP-2), the half-life of which is 16-fold longer that of the native GLP-2. This study confirmed that the GLP-2 analogue was able to lower the inflammation score in the affected colon and enhance the level of proinflammatory cytokines. A few human studies also confirmed the protective effect on IBD. Buchman et al. [100] designed a prospective placebo-controlled study in which different doses of teduglutide were administrated in 100 patients with Crohn’s disease for 8 weeks. They figured out that the ultimate remission rate of patients injected with high-dose teduglutide was higher than that in patients without teduglutide.

#### 5.1.3. DPP-4 Inhibitors

A few studies have shown that DPP-4 inhibitors have a protective effect on IBD. The mechanism, however, is not yet properly understood. Currently, the anti-IBD effect of DPP-4 has been mainly regarded as the result of the upregulated expression of GLP-1 and GLP-2 [105,106]. Detel et al. [101] took a closer look at the influence of DPP-4 deficiency on the course of colitis. They first adopted DSS administration to induce colitis in DPP-4-deficient mice and wildtype mice. They found that the myeloperoxidase activity in DPP-4-deficient mice was higher than that in wildtype mice through analysing inflamed colonic tissue, which was also in accord with the expression of the NF-κB p65 subunit. Salaga et al. [102] investigated the effect of EMDB-1 (a DPP-4 inhibitor) on colitis in a mouse model, which was demonstrated to ameliorate intestinal inflammation in mouse colitis models induced by DSS and TNBS. Currently, a very limited number of human studies have observed the therapeutic effect of DPP-4 on IBD patients. However, a couple of meta-analysis studies examined a correlation between the risk of developing IBD and DPP-4 inhibitor administration. Abrahami et al. [103] adopted a cohort study to analyse the influence of DPP-4 inhibitors on the incidence of IBD in type 2 diabetes patients, including 141,170 participants (age >18 years). They recognised that there was a correlation between DPP-4 inhibitor administration and an increased risk of IBD. In 2019 [104], another meta-analysis study involving 16 separate studies (198,404 patients in total) aimed at investigating the risk of developing IBD with DPP-4 inhibitor treatment. The author concluded that DPP-4 inhibitors do not appear to augment the risk of developing IBD. On the contrary, the animal studies suggest that DPP-4 inhibitor administration is able to reduce the level of colonic inflammation, which contradicts the conclusions found in human studies. To sum up, the effect and mechanism of DPP-4 regulating IBD are still unclear. More human studies and further experiments are necessary in order to explore the role of DPP-4 inhibitors in this regard.

### 5.2. Ghrelin and IBD

In the human body, enteroendocrine X cells produce and secrete ghrelin. As an enterohormone, ghrelin exerts multiple functions in the maintenance of homeostasis. In addition to the management of food intake, ghrelin has been demonstrated to reduce the intestinal inflammation in rodent models of IBD (see Table 3). As mentioned above, some varieties of flavonoids have the capacity to increase ghrelin secretion, whereby we infer that flavonoids may have an impact on IBD through modulating ghrelin secretion.

Gonzalez-Rey et al. [107] attempted to find out the therapeutic action of ghrelin in mice models of colitis induced by TNBS. In the rodent experiment, colitogenic mice were first induced to develop colitis through an intrarectal application of TNBS. Then ghrelin was administrated to colitis mice by intraperitoneal injection and compared with the control group, which was not affected by ghrelin. Finally, the authors analysed a set of parameters reflecting the activity of colonic inflammation. The results indicated that the administration of ghrelin significantly ameliorated weight loss and the histological colitis score, improved the survival rate and decreased the myeloperoxidase activity of colonic tissue in ghrelin-administrated mice in comparison with the control group. Furthermore, the authors also explored the possible anti-inflammatory mechanism of ghrelin (pro- and anti-inflammatory cytokines and chemokines). They found out that colonic TNF-α, IL-1β and IL-6 in ghrelin-administrated mice drastically decreased compared with the control group. Nonetheless, the level of colonic IL-10 (an anti-inflammatory cytokine) in ghrelin-treated mice increased up to two times higher than normal mice without colitis, which suggested that IL-10 contributed to the therapeutic benefits of ghrelin on colonic inflammation. Konturek et al. [108] examined the therapeutic effect of exogenous ghrelin on TNBS-induced colitis in rats. They found out that ghrelin significantly accelerated the healing of colitis induced by TNBS, which was related with the increased expression of iNOS and COX-2. In contrast to this, some studies drew the opposite conclusion—namely, that exogenous ghrelin aggravates colitis. Smet et al. [109] set up colitis models in mice through the oral administration of 3% DSS. Afterwards, the mice were injected with saline or ghrelin, respectively. The results showed that exogenous ghrelin increased the activity score of colitis, neutrophil infiltration in the colon and the expression of IL-1β, as well as the myeloperoxidase activity of colonic tissue in ghrelin-treated colitis mice compared with saline-treated colitis mice. Additionally, the authors compared the inflammatory level between wildtype mice and ghrelin knockout mice with DSS colitis. They drew the conclusion that there was a stronger tolerance of DSS colitis presented in ghrelin knockout mice in comparison with the wildtype mice. Obviously, the above two completely opposed conclusions need further studies involving in vivo and in vitro experiments. Recent evidence suggests that ghrelin takes part in the regulation of IBD through multiple pathways (see Figure 3B). For readers who are interested in the specific mechanism of ghrelin inhibiting IBD, please read the scientific paper written by Deboer [110].

### 5.3. CCK and IBD

The appearance of nutrients in the gut lumen can stimulate CCK release, which promotes the digestion of protein and fat [111]. Recently, some studies reported that CCK has an anti-inflammatory effect during in vivo and in vitro experiments, which is depicted in Table 4. Bozkurt et al. [112] injected CCK-8s (CCK Octapeptide) into colitis models of rats induced by acetic acid. The results indicated that all inflammation parameters, including the tissue wet weight index (WWI), histological score and myeloperoxidase (MPO) activity, increased in colitis rats in comparison with the control group; however, CCK-8 decreased these parameters. Therefore, CCK has an anti-inflammatory effect on colitis in rats. The lipid, enriching the enteral nutrition, is able to increase the level of CCK in circulation. Rodent and human studies have found that the administration of lipid-enriched enteral nutrition mitigated the inflammatory level in the intestine through relieving the enterocyte injury, decreasing the intestinal hyperpermeability and preventing bacterial displacement, as well as inhibiting the systemic inflammatory response [113,114]. In rat models induced by endotoxemia, Saia et al. [115] discovered that CCK pre-treatment decreased the mucosal production of cytokines stimulating inflammation formation, as well as increased the expression of seal-forming TJ proteins such as occludin, claudin-1 and the junctional adhesion molecule. These findings suggested that CCK could lower the level of the inflammatory response in the affected intestine and improve the physical barrier of the intestinal epithelial cells. These in vivo studies showed that CCK has the potential to treat IBD successfully through its anti-inflammatory properties, as well as through its capability of maintaining the intestinal barrier. There are, however, only a few studies that have investigated the effect of CCK on IBD patients.

Concerning the immunomodulatory effect of CCK, it was reported that CCK can inhibit the activation of dendritic cells (DCs) [116], which may be beneficial for the treatment of IBD. Recent studies have suggested that CCK-8 can regulate immune cells (T cells and B cells). Zhang et al. found that CCK-8 inhibited the differentiation of Th1 and Th17 and boosted the development of Th2 and Treg in vitro. Meanwhile, CCK8 decreased the expression level of costimulatory molecules like CD86 and CD80 in lipopolysaccharide-activated B cells in vitro [117,118]. Until now, the mechanisms of the CCK effects in suppressing IBD are still unclear. For the potential mechanisms, see Figure 3C. As mentioned above, flavonoids are truly capable of promoting the secretion of CCK, thus indirectly regulating IBD.

## 6. Conclusions

As an incurable disease, inflammatory bowel disease repeatedly occurs and thereby profoundly affects patients’ quality of life. Accumulating evidence has shown that the maintenance of intestinal homeostasis can be modulated by flavonoids and EECs, respectively. Meanwhile, some studies found out that the intestinal disorders of IBD can be regulated by specific enterohormones released by EECs. Therefore, there is a possible link between flavonoids, the enteroendocrine system and IBD. Over the past decades, most studies have focused on the treatment of flavonoids in IBD through their antioxidant property, thus protecting the intestinal physical barrier through their immunomodulatory property in the gastrointestine and through their capability at shaping the microbiota composition and function. Further studies on the basic functional role of flavonoids in IBD and their improved use in these processes could contribute to finding new successful therapeutic options for the treatment of IBD in the future.

## Figures and Tables

**Table 1:** Flavonoids: classification and food source classes.

Class	Forms Existed	Dietary Sources
Flavones	Apigenin, Chrysin, Luteolin, Baicalin	Buckwheat, Fruit peel, Tomato skin
Isoflavones	Daidzein, Genistein	Chinese herb, Soybeans
Flavanols	Epicatechin, Catechin	Chocolate, Tea, Fruits
Flavanonols	Taxifolin, Astilbin	Onion
Flavanones	Naringin, Hesperidin, Naringenin	Grape, Citrus fruits
Flavonols	Kaempferol, Fisetin, Quercetin, Myricetin	Red wine, Olive oil, Grapes
Anthocyanidins	Delphinidin, Malvidin, Cyanide, Pelargonidin	Berries, Vegetables, Red wine

**Table 2:** Modulation of IBD by GLPs and DPP-4 inhibitors.

Hormone	Stimulating Factor	Model	Effects	Reference
GLP-1	DSS	GLP-1R knockout colitis mice	Increased weight loss, disease activity and intestinal epithelial damage.	[91]
GLP-1	DSS	Colitis mice	No decrease in the level of intestinal inflammation.	[92]
GLP-1	CD4^+^CD25^−^ T cells	GLP-1R knockout colitis mice	Decreased the inflammatory score in histopathology and the level of proinflammatory cytokines.	[93]
GLP-1	DSS	Colitis mice	Decreased weight loss, histological destruction, improved stool consistency, reduced IL-1β and increased the expression of the intestinal chloride transporter.	[94]
GLP-1	Null	UC patient	Symptomatic remission of UC.	[95]
GLP-1	Null	IBD patients	Improved the process of IBD.	[13]
GLP-2	DSS	Colitis mice	Reduced IL-1 and increased the colon length, crypt depth and both mucosal area and integrity in the colon.	[96]
GLP-2	Null	IL-10-deficient colitis mouse	Decreased the inflammation score in histopathology and lowered the MPO, IL-1β, IFN-γ and TNF-α.	[97]
GLP-2	DSS	Colitis mice	Decreased weight loss and increased colon length.	[98]
GLP-2	DSS	Colitis rats	Decreased colonic damage score and expression of IL-1, IL-7 and TNF-α.	[99]
GLP-2	Null	CD patients	Induced remission and mucosal healing in CD patients.	[100]
DPP-4 inhibitors	DSS	DPP-4-deficient colitis mice	Increased MPO and expression of the NF-κB p65 subunit.	[101]
DPP-4 inhibitors	DSS and TNBS	Colitis mice	Increased GLP-2 and decreased MPO, weight loss and histological destruction.	[102]
DPP-4 inhibitors	Null	IBD patients	Increased risk of IBD.	[103]
DPP-4 inhibitors	Null	IBD patients	Did not augment the risk of IBD.	[104]

**Table 3:** Modulation of IBD by ghrelin.

Hormone	Stimulating Factor	Model	Effects	Reference
Ghrelin	TNBS	Colitis mice	Reduced weight loss, histological colitis score and MPO; increased IL-10 and decreased TNF-α, IL-1β and IL-6.	[107]
Ghrelin	TNBS	Colitis rats	Accelerated the healing of TNBS colitis and increased the expression of iNOS and COX-2.	[108]
Ghrelin	3% DSS	Colitis mice	Increased the activity score of colitis, neutrophil infiltration, IL-1β and MPO.	[109]

**Table 4:** Modulation of IBD by CCK.

Hormone	Stimulating Factor	Model	Effects	Reference
CCK	Acetic acid	Colitis rats	Decreased inflammation parameters (WWI, histological colitis score and MPO).	[112]
CCK	LPS	Sepsis mice	Relieved intestinal epithelium damage and prevented bacterial displacement.	[113]
CCK	LPS	Healthy men	Decreased TNF-α, IL-6, IL-1 and increased IL-10.	[114]
CCK	LPS	Sepsis rats	Decreased TNF-α, IL-1ß, prevented bacterial displacement and increased tight junction.	[115]
CCK	CpG ODN	Dendritic cells	Decreased IFN-α and inhibited TNF receptor-associated factor 6.	[116]
CCK	LPS	B cells	Inhibited CD86 and CD80.	[117]
CCK	Null	T cells	Inhibited Th1 and Th17 and boosted Th2 and Treg.	[118]
